# Supplementary material for: The exceptionally high diversity of small carnivorans from the Late Miocene hominid locality of Hammerschmiede (Bavaria, Germany)
Source: PLoS One. 2022 Jul 13;17(7):e0268968. doi: 10.1371/journal.pone.0268968 (PMC9278789; doi:10.1371/journal.pone.0268968)
Supplement: Attachment — Submitted filename: Changes List.docx [file pone.0268968.s002.docx]

To the Editorial Board and the Reviewers,

We would sincerely like to thank you for reviewing our manuscript and for your comments that have helped improving it. Below, there is a list of the changes we have made, attributed to the Editor, Reviewer 1, Reviewer 2 and Personal Changes. The page (P) and line (L) number correspond to that of the initially submitted PDF version starting from the title page.

**Editor**

P.1 L.7 to P.2 L.24: The affiliations have been corrected in order to match the journal’s requirements.

P.2 L.29 etc.: The main titles have been changed to 18pt font.

P.4 L.87: The sentence: “No permits were required for the described study, which complied with all relevant regulations” has been added.

P.5 L.95: The relevant paragraph concerning the Nomenclatural Act has been added in Material and Methods.

P.21 L.399: The LSID for the new species has been added.

**Reviewer 1**

P.3 L.55: A comma has been added after “molluscs”.

P.4 L.74: The sentence “More information about the geographic position and the stratigraphy of the locality can be found in [1, 16]” has been added at the end of the paragraph. This has been chosen over the inclusion of new figures, because these data have already been published in the respective articles and copying them in the present manuscript will raise the need of asking for copyrights’ permission to use them.

P.4 L.75: A comma has been added after “(11.44 Ma)”.

P.4 L.81–82: The respective phrase has not been deleted as it supports our suggestion for the lateral correlation of HAM 1 to HAM 5.

P.4 L.86: A comma has been added after “(GPIT)”.

P.8 L.160–161: The measurements of the incisors have been added to a table (Table 2). All the Tables’ numbering has been changed after that.

P.8 L.196: As stated in P.8 L.195, these measurements are already in former Tables 2 and 3 (New Tables 3 and 4).

P.12 L.249 and P.19 L. 362: The word “carnassials” has been corrected.

P.12 L.252: The word “differences” has been corrected.

P.12 L.254: The word “more” has been replaced by “stronger”.

P.12 L.259: The phrase “the skull of the” has been added before “extant species”.

P.15 L.292: The figure references are mentioned in the descriptions.

P.15 L.303 etc.: The terms “cingulum”, “cusp” and “crista” have been corrected as “cingulid”, “cuspid” and “cristid” for the lower teeth.

P.16 L.313 and P.23 L.443: The respective views have been added in the figure caption.

P.16 L.318: The phrase “most specimens of” has been added before “*M*”. *munki*.

P.17 L.347 etc.: We accept the suggestion of the reviewer to refer to this form as “*M*”. cf. *munki*. This has been changed throughout the text.

P.19 L.370: The word “any” has been deleted.

P.21 L.409: The measurements for *Circamustela hartmanni* are already in the Tables 4 and 5.

P.39 L.768: The phrase “upper molar” has been replaced by “M1”.

P.39 L.774: The phrase “lower fourth premolar” has been replaced by “p4”.

P.51 L.1001: No measurements have been taken in the described braincase as it is too damaged to preserve clear measuring points.

P.55 L.1078 and P.83 L.1606: The name “*Rhodanictis*” has been changed into “*Trochictis depereti*”.

P.69 L.1350: More comments have been added supporting the attribution of these specimens to the genus *Potamotherium*. Figure 23 has been expanded presenting comparative material. Dr S. Mayda that provided one of these photographs has been added to the acknowledgments. The numbering of the literature has been changed accordingly.

P.107 L.2137 and P.114 L.2293: The references have been corrected.

**Reviewer 2**

P.21 L.403: The phrase “Hartmann family” has been changed into “Antonie Hartmann”. This resolves the problem of the name of the new species.

P.20 L.379: A figure presenting two scatter plots comparing the M1 and m1 of the “*Martes*” material from Hammerschmiede to other members of the genus has been added.

P.26 L.500: A figure presenting two scatter plots comparing the P4 and m1 of *Circamustela* *hartmanni* to *C*. *peignei*, *C*. *dechaseauxi* and *Aragonictis araid* has been added.

P.84 L.1620: A figure presenting the stratigraphical range of the discovered species has been added.

The reviewer suggested that the addition of a PCA for the skulls of “*Martes*” *sansaniensis* and a quantitative approach on the ecomorphology of the discussed carnivorans would be useful. We agree to this point. However, both cases are parts of two separate projects: the former concerns the exact taxonomic position of the Miocene “*Martes*” in regard to the extant *Martes* and the latter concerns the study of the carnivoran guild of Hammerschmiede as a whole. Therefore, even though we consider that this comment has merit, we would prefer to present these data in their broader frame in the relevant projects.

**Personal Changes**

P.14 L.287: The sources of data have been corrected and the locality “Sant Quirze” has been changed into “Sant Quirico”.

P.25 L.481: The authors took themselves new measurements in the holotype of *Circamustela dechaseauxi*. The new measurements have replaced the original of Petter (1967) in the relevant table.

P.31. L.607 etc.: During the weeks that the review process took place, the publication of Alba et al. about *Eomellivora* *moralesi* has been published in printed version. Therefore, the authorship has been changed into Alba et al., 2022 in all the relevant points of the manuscript.

P.46 L.895: The measurements for *Paralutra jaegeri* from Steinheim have been corrected.

P.58 L.1139: The measurements for *Proputorius pusillus* have been corrected.

P.82 L.1590: The word “Sc” has been corrected into “SF”.

P.91 L.1764: The sentence “The authors would like to thank Dr. M. Morlo and one anonymous reviewer, as well as the editorial board of PLoS ONE for their fruitful comments” was added.

Best regards,

Nikolaos Kargopoulos
